# Case Report: Long-Term Treatment and Follow-Up of Kleefstra Syndrome-2

**DOI:** 10.3389/fped.2022.881838

**Published:** 2022-05-24

**Authors:** Dandan Wu, Rong Li

**Affiliations:** Child Healthcare Department, Children's Hospital of Nanjing Medical University, Nanjing, China

**Keywords:** Kleefstra syndrome-2, *KMT2C*, children, ADHD, follow-up

## Abstract

**Background:**

Mutations in the *KMT2C* gene can cause Kleefstra syndrome-2 (KLEFS2).

**Case:**

In this study, we analyzed the clinical, genetic testing, and 10-year follow-up data of a child with KLEFS2 treated at the Child Healthcare Department, Children's Hospital of Nanjing Medical University, Nanjing. The case of KLEFS2 presented feeding difficulty and developmental delay, both intervened by nutritional support and family rehabilitation. Obvious attention deficit hyperactivity disorder (ADHD) occurred in preschool and school-age children and was managed by behavioral and pharmaceutical interventions.

**Conclusion:**

Features of KLEFS2 include feeding difficulty and developmental delays in an early age, as well as ADHD in preschool and school age. Satisfactory outcomes are not achieved in early nutritional support for correcting malnutrition and pharmaceutical intervention for relieving ADHD, but both measures can counter developmental delay.

## Introduction

Kleefstra syndrome (KLEFS) is a rare autosomal dominant disease characterized by abnormal facial appearance, language and motor delays, intellectual disability, and childhood hypotonia, and some patients may present epilepsy, gastroesophageal reflux disease, and sleep disorders (1, 2). KLEFS is classified into Kleefstra syndrome-1 (KLEFS1, OMIM: 610253) caused by terminal deletions of chromosome 9q34.3 or mutations of the Euchromatic Histone Lysine Methyltransferase 1 (*EHMT1*) gene (3, 4) and Kleefstra syndrome-2 (KLEFS2, OMIM: 617786) caused by mutations of the *KMT2C* gene (5). KLEFS1 was initially described in 1999. At present, there are more than 100 cases of KLEFS1 worldwide. Only 7 cases of KLEFS2 have been reported so far, but all lack long-term follow-up data. In the present case report, we reviewed the clinical manifestations and follow-up data of a case of KLEFS2 from 9 months to 10 years of age and analyzed the pathogenicity of frameshift mutations of the *KMT2C* gene, aiming to elucidate its therapeutic strategy, clinical outcome, and prognosis.

## Clinical Data

### Baseline Characteristics

This male infant was the second full-term birth delivered through cesarean section, with a birth weight of 2.5 kg and a height of 50 cm. His parents were not consanguineously married and the mother's pregnancy history was normal. All his parents and sibling were healthy. At the age of 9 months, the patient was taken to the Child Healthcare Department several times for feeding difficulty, comprehensive developmental delay, anemia, and eczema. From the age of 5 years and

**Figure 1:**
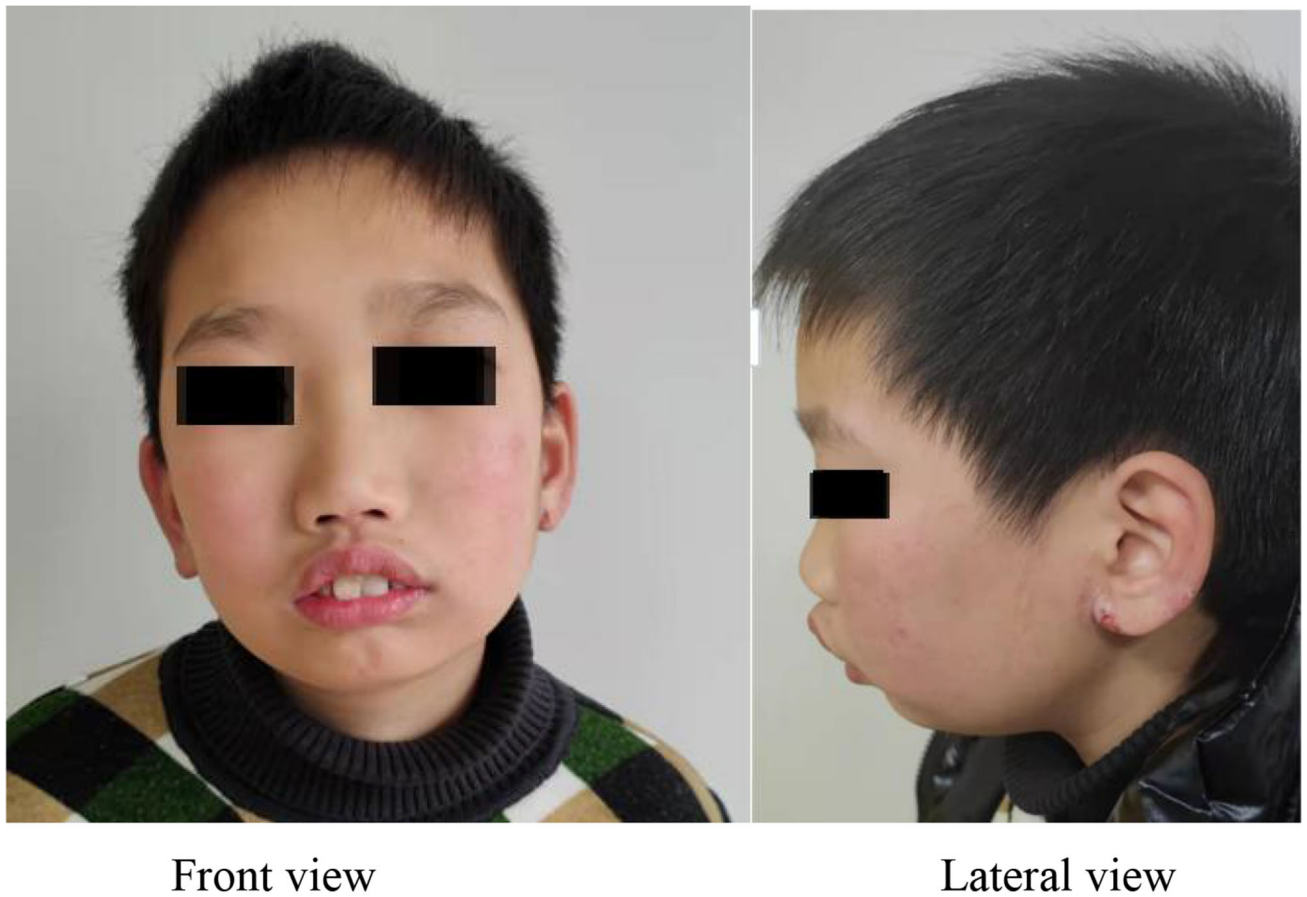
Facial features of the patient, such as bushy brows, mandibular retrusion, and ear eczema.

5 months, the patient was treated in our center for obvious attention deficit hyperactivity disorder (ADHD) symptoms. Physical examinations showed distinctive facial features, such as bushy brows, mandibular retrusion, and ear eczema (Figure 1). Cardiopulmonary palpation was normal, and subcutaneous fat in the abdomen was rich. The spine was located in the midline without scoliosis. Muscle tension and strength of extremities were normal. The body skin was dry.

### Treatment

#### Physical Development Assessment

**Figure 2:**
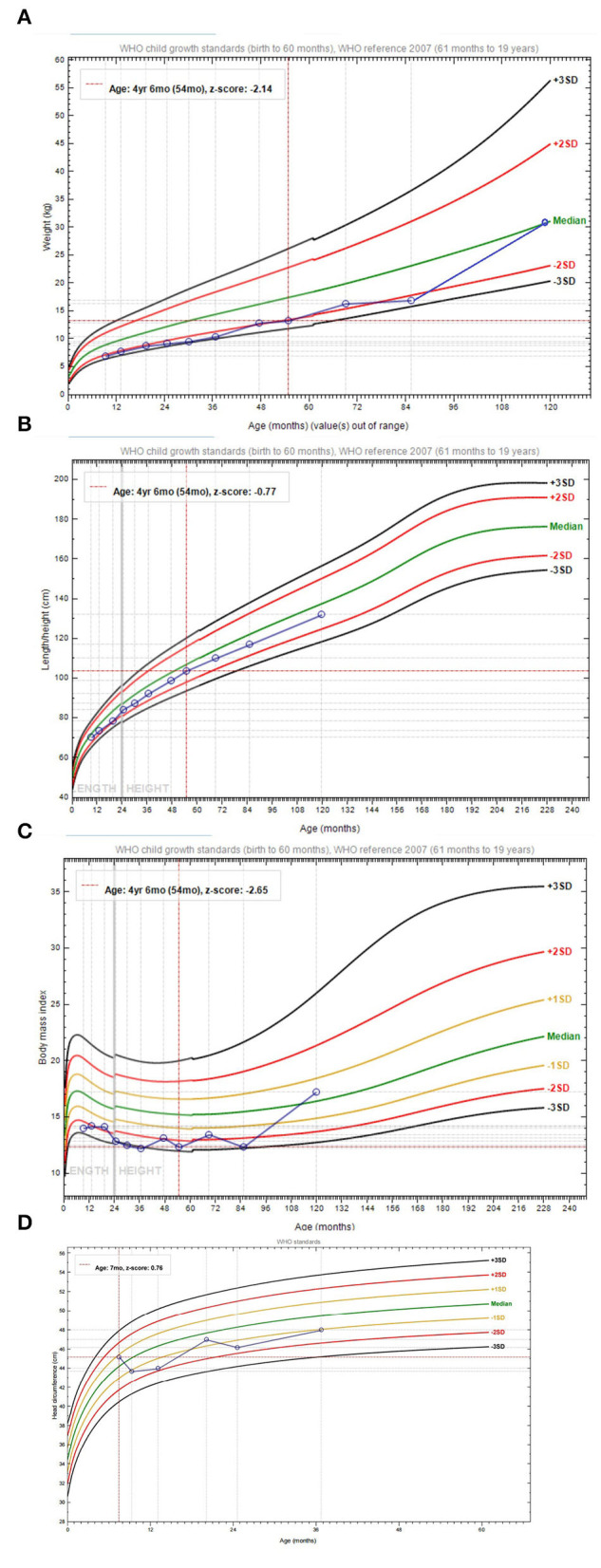
The growth curve. **(A)** The body weight curve (0–10 years). **(B)** The height curve (0–10 years). **(C)** Body mass index curve (0–10 years). **(D)** Head circumference curve (0–3 years).

At the age of 9 months, the patient presented at the Child Healthcare Department for feeding difficulty, comprehensive developmental delay, anemia, and eczema. Interventions with an amino acid-based high-energy formula and a high-energy high-protein diet exerted a positive effect on emaciation and developmental delay, but not on ear eczema. At the age of 7 years and 3 months, the patient was diagnosed with ADHD with weight loss and was treated with methylphenidate (CONCERTA^®^). The patient was presented to Nanjing Brain Hospital at the age of 9 years for ADHD again. After starting aripiprazole medication, his body weight increased rapidly (Figure 2; Table 1).

**Table 1:** Follow-up data of the patient with Kleefstra syndrome-2 (KLEFS2) from 9 months to 10 years.

**Event**	**Treatment/testtime**	**Interventions/test items**	**Outcomes**
Difficult feeding, developmental delay and eczema	9 months to 6 years	Amino acid-based high-energy formula and high-energy high-protein diet	Emaciation and developmental delay were inconspicuously alleviated; Eczema still existed.
	6 to 8 years	High-energy high-protein diet	Emaciation, developmental delay and eczema were not alleviated.
Anemia	9 months to 1 year	Ferrotherapy for 3 months	Anemia was not obviously alleviated. Mild β-thalassemia was diagnosed.
Comprehensive developmental delay	1.58 years	Children's Developmental Center of China (CDCC)	MDI: 63, PDI <50
	3.25 years	Peabody Picture Vocabulary Test (PPVT)	IQ:80 points
	4.75 years	Wechsler Preschool and Primary Scale of Intelligence (WPPSI-II)	FIQ:90 points
	7.1 years	Wechsler Preschool and Primary Scale of Intelligence (WPPSI-II)	FIQ:97 points
	10.5 years	Wechsler Intelligence Scale for Children (WISC-IV Chinese version)	FIQ:87 points
Attention deficit	1.58 years	Neglected by parents.	
	5.42 years	Parents were trained about interventions and parenting skills	Attention deficit was improved.
	7.1 to 7.25 years	20 mg/d atomoxetine hydrochloride	Irascibility and impulse behaviors.
	7.25 to 8.1 years	18 mg/d atomoxetine hydrochloride	Hyperactivity symptoms were improved, and attention deficit was not obviously improved. Loss of body weight of 2 kg within 10 months.
	9 to 10 years	1.25 mg/d aripiprazole	Leaving a seat in the classroom was corrected, and impulsive behaviors were improved. Body weight increased by 14 kg per year, especially the gain of abdominal fat.
	10.42 years	18 mg/d methylphenidate hydrochloride	Impulsive behaviors were improved. The body weight was followed up.
Kleefstra syndrome-2	10.42 years	Being followed up	

*MDI, mental development index; PDI, physical development index; IQ, verbal intelligence quotient; FIQ, full-scale intelligence quotient*.

#### Developmental Assessment

Developmental assessment of this patient showed a comprehensive developmental delay at an early age, which accelerated later (Table 1).

#### Behavioral Assessment

The patient poorly cooperated and was easily distracted during the behavioral assessment and interactive process, which were not considered seriously by his parents. His hyperactivity and attention deficit were reported by his kindergarten teachers at the age of 5 years and 5 months, and he was finally diagnosed with ADHD. Psychiatrists interviewed the child and his parents and made the clinical diagnosis using the diagnostic criteria of ADHD in Diagnostic and Statistical Manual of Mental Disorders, Fifth Edition (DSM-V). When we trained his parents to perform behavioral interventions and parenting skills, the hyperactivity symptoms of the patient were alleviated. However, his ADHD symptoms aggravated at the age of 6 years, and atomoxetine hydrochloride was given at the age of 7 years and 1 month, with an initial dose of 0.5 mg/kg/day and a target dose of 1.2 mg/kg/day. After a 2-month medication, ADHD symptoms were not alleviated, and the patient developed irascibility and impulsive behaviors. Methylphenidate at the dose of 18 mg/day was given at the age of 7 years and 3 months, and it significantly alleviated hyperactivity symptoms. Inattention symptoms, however, were not remarkably improved. The patient's appetite was low and his body weight decreased by 2 kg during the 10 months of methylphenidate medication, which was therefore withdrawn by his parents intentionally. At the age of 8 years and 1 month, the patient showed serious violations against classroom rules, and he was again presented to the Nanjing Brain Hospital at the age of 9 years for ADHD. Medication of 1.25 mg/day of aripiprazole could keep him seated in the classroom at the early stage but could not remarkably correct attention deficit and hyperactivity symptoms. His body weight increased by 14 kg after 1 year of aripiprazole medication, especially the high gain in abdominal fat. Considering the well-controlled ADHD symptoms, aripiprazole was withdrawn by his parents. However, his parents did not perform any behavioral interventions during the 5 months after withdrawal, and the patient could not stay seated in the classroom again or even ran away from home. At the age of 10 years and 5 months, the patient was represented to our center for medical management. Medication of 18 mg/day methylphenidate significantly alleviated his behavioral disorders in the classroom and corrected other hyperactive and impulsive behaviors to some extent (Table 1).

#### Assessment of Autism Spectrum Disorder

An assessment of autism spectrum disorder (ASD) was performed in the present case at the age of 10 years and 6 months. The Autism Behavior Checklist (ABC) score of 26 points and the Childhood Autism Rating Scale (CARS) score of 23 points both indicated the absence of ASD.

#### Other Examinations

Both the blood routine test and the thyroid function test at the age of 9 months were normal. A computed tomography (CT) scan of the brain showed no dilation of bilateral lateral ventricles, the low position of the cerebellar tonsil, enlargement of cisterna magna, and small sella turcica.

### Genetic Testing

At the age of 9 months, a 3-month ferrotherapy was given to this patient for his low level of hemoglobin, but the clinical outcome was unsatisfactory. The next-generation sequencing (NGS) using the GenCap Platform revealed mutations of c.316-197C>T in the *HBB* gene, confirming the diagnosis of mild β-thalassemia. The mutation site was subsequently identified to be inherited from his father. Then, at the age of 10 years, the whole-exome sequencing (MyGenostics, Beijing, China) revealed NM_170606 (*KMT2C*): c.9284delC (p.P3095Lfs^*^2), suggesting a frameshift mutation. Pedigree verification identified it as a *de novo* mutation. According to the evidence-based criteria of the American College of Medical Genetics and Genomics (ACMG), the NM_170606 (*KMT2C*): c.9284delC (p.P3095Lfs^*^2) was a pathogenic frameshift mutation (PVS1 + PS1 + PM2_Supporting), which may result in a loss of function (LOF) effect (PVS1). It was a *de novo* mutation (PS1), which was not detected in his parents. In addition, this novel mutation has not yet been reported in any database (PM2_Supporting).

## Discussion

Kleefstra syndrome, caused by the haploinsufficiency of *EHMT1*, is characterized by intellectual disability, ASD, characteristic facial dysmorphisms, and other variable clinical features. In addition to *EHMT1* mutations, *de novo* variants have been reported in four additional genes (*MBD5, SMARCB1, NR1I3*, and *KMT2C*). Although KLEFS1 cases have been reported, the number of KLEFS2 cases are rare and related long-term follow-up data are scant. This study reported a case of KLEFS2, providing a complete narrative of clinical features, genetic testing results, and long-term treatment and follow-up.

Koemans et al. (5–8) summarized the common features of seven patients with KLEFS2 aged 7–31 years, such as mild to severe intellectual disability, language/motor development delay, and behavioral disorders (e.g., autism spectrum disorder, ADHD, sleep problems, self-injurious behavior, and aggressive behavior). To note, three patients with KLEFS2 developed hypotonia and two developed epilepsy in childhood. Scoliosis, short stature, microcephaly, recurrent respiratory infections, phenylketonuria, hypospadias, dry skin, and hoarseness were reported in these cases as well. In the present report, the patient also developed language/motor development delay, ADHD, and dry skin. In addition, obvious feeding difficulty, persistent weight loss, and repeated eczema were not reported in previous cases. Notably, his physical development was improved to the normal level at the age of 3 years, but his intellectual development remained retarded.

In the present case, the nutritional intervention did not reverse weight loss after birth due to feeding difficulty and emaciation. Through reviewing his disease course and genetic testing data, we suggest that feeding difficulty and emaciation may be early-stage manifestations of KLEFS2, and patients with KLEFS2 may not benefit from nutritional interventions. Providing aripiprazole medication significantly increased body weight, especially the gain of abdominal fat. Nevertheless, this medication did not remarkably increase the height. Considering the close link between abdominal fat and visceral fat, the risk of long-term metabolic syndrome in KLEFS2 children should be well estimated. During the follow-up period, the liver function and the blood lipid profile of this case were normal, but the potential influence of KLEFS2 on metabolic diseases should be further evaluated.

Both KLEFS1 and KLEFS2 are caused by genetic defects involving histone methyltransferases. *KMT2C*, pathogenic gene of KLEFS2, is located on human chromosome 7q36. It encodes a histone methyltransferase that regulates gene transcription by altering chromatin structure. *KMT2C* induces transcriptional activation *via* mediating H3K4me1 and H3K4me3. A previous study has shown that the neuropathological mechanism of *EHMT1* (KLEFS1) mutations in Drosophila is similar to those of *KMT2C* (KLEFS2). There is a molecular convergence between the *KMT2C/D* homolog TRR and the *EHMT1* homolog G9a; this convergence plays a decisive role in gene regulatory networks responsible for normal neuronal development and function, especially for complex brain functions, such as learning and memory (5). In addition, four of the five potential target genes of *TRR*, such as *REG-2, Acer, PCB*, and *Arc-1*, are directly or indirectly implicated in the formation of memory. Among them, *REG-2* and *Acer* can regulate circadian rhythm (9, 10). *Arc* is an immediate-early gene that is activated in neurons associated with learning and memory (11). Memory is a basic element of intelligence. In the present case report, the frameshift mutation in the *KMT2C* gene may cause an LOF effect, thus impairing his learning and memory. In addition, the intelligence of this case might be underestimated due to attention deficit, a condition arising from comprehensive developmental delay. Notably, the intelligence of this case gradually returned to normal with aging, suggesting that patients with KLEFS2 may be exempted from intellectual disability after a long term.

In this case, severe ADHD symptoms significantly influenced his sociability, and he was unable to study attentively in the classroom. Low-dose atomoxetine hydrochloride, methylphenidate hydrochloride, and aripiprazole did not remarkably alleviate ADHD symptoms, but induced adverse events, such as irascibility, appetite loss, and body weight fluctuation. It is suggested that patients with KLEFS2 may not be sensitive to ADHD medications. Impulsive behaviors and attention deficit, in this case, were significantly improved at the age of 5 years through behavioral interventions performed by his parents but aggravated again after these interventions were interrupted. Therefore, behavioral interventions should be regularly given for a long period until the patient with KLEFS2 grows up, which is challenging and requires support from the family and the society.

Dry skin has been reported in KLEFS2 cases. In addition to dry skin, the present case also suffered persistent ear eczema and an increased eosinophil count, which have never been reported in previous cases. In the present case, electroencephalography findings were normal and epileptic seizures were not detected. The patient should be further followed-up for other manifestations, such as epileptic seizure, hypotonia, and scoliosis.

During the 10-year follow-up from 9 months to 10 years in this case, his pathological growth was difficult to be intervened with and his severe ADHD symptoms significantly disrupted his development. For those who only presented developmental delay without specific facial features, KLEFS2 was easily misdiagnosed because genetic testing needs time. Therefore, genetic testing should be timely carried out for pediatric patients with early-stage feeding difficulties, weight loss, repeated eczema, distinctive facial features, and ADHD symptoms insensitive to medications.

## Data Availability Statement

The original contributions presented in the study are included in the article/supplementary material, further inquiries can be directed to the corresponding author/s.

## Ethics Statement

The studies involving human participants were reviewed and approved by Ethics Committee of Children's Hospital of Nanjing Medical University. Written informed consent was obtained from the participant's legal guardian for the publication of this case report. Written informed consent was obtained from the participant's legal guardian for the publication of any potentially identifiable images or data included in this article.

## Author Contributions

RL designed the study and helped in writing the manuscript. DW performed the experiments, interpreted data, and wrote the manuscript. All authors contributed to the article and approved the submitted version.

## Funding

This work was supported by the Open Fund Program of Jiangsu Population Association (JSPA2019019).

## Conflict of Interest

The authors declare that the research was conducted in the absence of any commercial or financial relationships that could be construed as a potential conflict of interest.

## Publisher's Note

All claims expressed in this article are solely those of the authors and do not necessarily represent those of their affiliated organizations, or those of the publisher, the editors and the reviewers. Any product that may be evaluated in this article, or claim that may be made by its manufacturer, is not guaranteed or endorsed by the publisher.
